# Spatial and Multilevel Analysis of Unscheduled Contraceptive Discontinuation in Ethiopia: Further analysis of 2005 and 2016 Ethiopia Demography and Health Surveys

**DOI:** 10.3389/fgwh.2023.895700

**Published:** 2023-03-07

**Authors:** Koku Sisay Tamirat, Solomon Gedlu Nigatu, Getayeneh Antehunegn Tesema, Malede Mequanent Sisay, Zemenu Tadesse Tessema

**Affiliations:** Department of Epidemiology and Biostatistics, Institute of Public Health, College of Medicine and Health Sciences, University of Gondar, Gondar, Ethiopia

**Keywords:** contraceptive discontinuation, spatial distribution, Ethiopia, multilevel, EDHS

## Abstract

**Background:**

Unscheduled discontinuation of contraceptives is a public health problem among women of reproductive age. Particularly, it is associated with unwanted pregnancies that lead to maternal and child mortality, but little is known about the spatial distribution of the problem. Therefore, this study aims to assess the spatial distribution and associated factors of unscheduled contraceptive discontinuation in Ethiopia.

**Method:**

This study used secondary data from the Ethiopia Demography and Health Survey (EDHS) data of 2005 and 2016. The study population was women who used contraceptives in the preceding 5 years before the survey. A total of 2,327 and 3,858 eligible women were included in the final analysis of the 2005 and 2016 EDHS, respectively. For the spatial analysis, both the 2005 and the 2016 EDHS data were analyzed using ArcGIS version 10.7, while for multilevel regression analysis, the 2016 EDHS data were used. The final model reported an adjusted odds ratio (AOR) with a 95% confidence interval (CI), and a *p*-value of 0.05 was used to declare statistical significance.

**Result:**

This study revealed that unscheduled discontinuation of contraceptives varied geographically, and hotspots were detected in the central, north, and eastern parts of Ethiopia. Moreover, diploma and higher education (AOR = 1.40; 95% CI: 1.01–1.95), urban residence (AOR = 1.37; 95% CI: 1.08–1.72), history of termination of pregnancy (AOR = 1.47; 95% CI: 1.14–1.94), married women (AOR = 10.79; 95% CI: 6.98–16.69), separated/divorced women (AOR = 1.54: 95% CI: 1.07–2.30), —two to four number of children (AOR = 1.46; 95% CI: 1.15–1.84), and involvement in the decision-making process of contraceptive use (AOR = 39.26; 95% CI: 28.84–53.45) were all factors associated with unscheduled discontinuation of contraceptives.

**Conclusion:**

This study revealed that unscheduled discontinuation of contraceptive distribution was significantly clustered in the central, north, and eastern parts of Ethiopia, as found in two surveys. The magnitude of this discontinuation increased from 2005 to 2016. The finding underscores that further interventions such as the availability of multiple mixed methods and improvement in women's decision-making ability in the choice of contraceptive methods and utilization are needed in hotspot areas of Ethiopia.

## 1. Background

Family planning (FP) methods contribute to desirable birth spacing, a control of the number of children that one can have, and improvement in maternal and child health (1). Improved and sustained use of contraceptive methods contributes to child survival rates, reduces the rates of unwanted and unplanned pregnancies and unsafe abortions, and improves maternal wellbeing. More specifically, increased coverage of family planning services has contributed to the reduction of maternal mortality rates in Ethiopia and the achievement of the United Nations Millennium Development Goal (2). Furthermore, increased contraceptive use among women of reproductive age has a positive impact on women’s social and economic participation, engagement, and empowerment (3).

Most users of contraceptives prefer short-term methods such as pills and injectables to long-term methods, which may lead to a problem of adherence. Still in Ethiopia, millions of unwanted and unplanned pregnancies as well as unsafe abortions have been reported as a result of unscheduled discontinuation and an unmet need for contraceptives. Unscheduled discontinuation is defined as the starting and stopping of contraceptive use while the woman is at risk of pregnancy and who has no intention of remaining fertile (4). Unscheduled discontinuation of contraceptives is one of the public health problems among reproductive-age women. Discontinuation of contraceptives is associated with unbearable maternal and child health, morbidity, and mortality through increased unwanted pregnancies (3, 5, 6). A study in Thailand showed that the incidence of contraceptive discontinuation ranged from 21.3 per person years of combined oral contraceptive pills (COCs) to 2.3 per person years of implants, and approximately 83.3% of women discontinued contraceptive use because of side effects (7). Another study in Kenya showed that the rate of contraceptive discontinuation increased in urban areas from 27.7% to 48.9% from the years 2003 to 2014, given that condoms and pills were the most common methods of discontinuation (4).

Poor counseling, side effects, and limited accessibility of health services cause increased discontinuation of contraceptives. In addition, sexual partner disapproval, distance to health facilities, a lack of joint decision-making, and religious and cultural taboos against the use of contraceptive methods are some of the reasons for the discontinuation of contraceptives (4, 8–10).

Although a few studies have been conducted to identify the factors associated with contraceptive discontinuation, evidence regarding the geographical distribution of the problem is scarce in Ethiopia. Therefore, this study aims to assess the spatial distribution and determinants of unscheduled contraceptive discontinuation in the country. The findings could give an insight into the geographical distribution and hotspots of the problem and support targeted interventions.

## 2. Methods

### 2.1. Data sources and study setting

The secondary data from the 2005 and 2016 Ethiopia Demography and Health Surveys (EDHSs) were downloaded from the measure DHS website at www.dhsprogram.com, following online request approval and permission. Ethiopia is the second largest populous country in Africa, with the majority of the population living in rural areas, and people’s livelihood depends on agricultural activities. In addition, the country has nine regional states and two city administrations.

### 2.2. Study population and data extraction procedure

The EDHS used a stratified two-stage cluster sampling, where regions were initially stratified into urban and rural areas. The enumeration areas (EAs) and households were used as primary and secondary sampling units and were selected independently in each stratum in two stages. The full EDHS report presents the detailed sampling procedure (11). The study population for this study is women who ever used contraceptives in the preceding 5 years before the 2005 and 2016 surveys. Thus, women who used contraceptives and discontinued planned pregnancies and those on whom no latitude and longitude data were available were excluded from the study. After extraction, the final sample size was 2,327 for the 2005 EDHS survey and 3,858 for the 2016 one.

### 2.3. Measurements of variables

Unscheduled discontinuation of contraceptives among reproductive-age women who have been using contraceptives, as found in the two surveys, was the outcome variable. Thus, women who had a history of unplanned discontinuation of contraceptives for the last method were coded as “1” and those who had no such history were coded as “0” in both DHS data (12).

### 2.4. Independent variables

Individual-level variables were as follows: respondents’ age, marital status, level of education, media exposure, awareness about family planning, healthcare access problems, wealth status, sex of household head, history of termination of pregnancy, and the number of children were explanatory variables. However, place of residence (either urban or rural areas) was also a community-level independent variable for this study.

### 2.5. Data processing and analysis

#### 2.5.1. Spatial analysis

Latitude and longitude coordination were combined with the Ethiopian shapefile (downloaded from GADM.org) using a spatial location to obtain the region's name for each cluster. The incremental spatial autocorrelation method was used to identify appropriate distance band or distance radius parameters by measuring the spatial autocorrelation for a series of distances and identifying significant peak *z*-scores that indicate distances where spatial processes promoting clustering are the most pronounced (13). Global Moran's I was used to assess the presence of the spatial autocorrelation (global pattern) of unscheduled contraceptive discontinuation in Ethiopia. However, both Anselin local Moran's I and Gets Ord-Gi* were used to assess for local clustering and outliers within the study area (13, 14). Although many interpolation techniques are available to predict values for unsampled areas from a limited number of sample data points (14, 15), the Empirical Bayesian Kriging (EBK) estimate was used for this study. It provides both a straightforward and a robust method of data interpolation by accounting for the error introduced by estimating the semi-variogram model. The spatial scan statistical method was used; this is the widely recommended method because of better local cluster detection and higher power than other available spatial statistical methods.

#### 2.5.2. Spatial scan statistical analysis

A spatial scan statistical analysis is paramount to identifying the most significant clusters of unscheduled contraceptive discontinuation across Ethiopia. We applied Bernoulli-based model spatial scan statistics since the outcome variable is dichotomous, and we used Kuldorff's SaTScan version 10.1 software. Statistically significant spatial cluster windows were identified using a *p*-value < 0.05 and likelihood ratio tests.

#### 2.5.3. Multilevel regression analysis

To restore representativeness from disproportionate sampling and non-response, sample weighting was applied to individual interview units during analysis. A multilevel model was used to account for the hierarchical nature of EDHS data where individual-level variables are nested within selected enumeration areas. In addition, an intraclass correlation (ICC) value of 0.21 and the statistically significant LR test were the null model, indicating that the multilevel model is better than traditional logistic regression. A less than 0.2 *p*-value during bivariable multilevel binary logistic regression analysis was used to select variables to be included in the final multivariable model.

Four models (null, individual-level, community-level variables, and both individual and community-level variables in the mixed-effect model) were compared using a log-likelihood (LL), and the model with the highest LL was selected as the best model fit. The equation for multilevel multivariable logistics regression can be presented as $\mathrm{Logit}(\;\pi_{ij})=\beta_{0}+\beta x_{ij}+U_{j}$, where *β*_0_ is an intercept, *β* is an unknown parameter for individual-level predictors, and *U_j_* (mean 0 and variance $\sigma_{u}^{2}$) are mutually independent Gaussian random effects.

The ICC is a ratio of the between-cluster variance to the total variance and was calculated as $ICC=V_{A}/(V_{A}+V_{I})=$ $V_{A}/(V_{A}+\pi^{2}/3)=V_{A}/(V_{A}+3.29)$. It shows the proportion of the total variance in the response variable that is accounted for by the cluster or correlation among observations within the same cluster and helps compare the successive models by considering the decline of the ICC. In addition, proportional change in variance (PCV) was also computed for each model concerning the empty model to show how much variability in the odds of anemia was explained by the successive models. The PCV was calculated as $\mathrm{PCV}=\frac{\left(V_{e}-V_{mi}\right)}{V_{e}}\times 100$, where *V_e_* is the variance in the empty model and *V_mi_* is the variance in successive models.

The median odds ratio (MOR), the median value of the odds ratio between the area at the highest risk and the lowest risk when randomly picking out two areas, was also computed to show the increase in the median risk that would occur if pregnant women in the low-risk area move to another area with a higher risk. This was calculated as $\mathrm{MOR}=e^{\left[\sqrt{(2*V_{A})0.6745}\;\right]}=e^{\left[0.95*\sqrt{V_{A}}\;\right]}$, where *V_A_* is an area-level variance for the particular model.

Multicollinearity was also checked to assess the correlation between independent variables. Explanatory variables with a high correlation with other independent variables were excluded from the multivariable multilevel logistic regression model. Finally, the adjusted odds ratio (AOR) with a 95% confidence interval (CI) was reported for variables with less than a *p*-value of 0.05 in the multivariable model.

## 3. Result

### 3.1. Sociodemographic characteristics

For this study, 2,327 and 3,858 women from 2005 and 2016 EDHSs were included in the final analysis, respectively. The median age of women was 30 years [interquartile range (IQR): 25–37] for the 2005 survey and 30 years (IQR: 24–36) for the 2016 one. The rate of women who attended primary school increased from 23.3% in 2005 to 33.6% in 2016. Moreover, 76.4% of women in 2005 and 59.5% in 2016 enjoyed the highest status of wealth. The two surveys found that 74.7% of households in 2005 and 76.4% in 2016 were headed by males. Likewise, 72.4% of women in 2005 and 56.3% in 2016 had media exposure (Table 1).

**Table 1:** Sociodemographic characteristics of women in Ethiopia in the years 2005–2016.

Characteristics	EDHS 2005, *n* (%)	EDHS 2016, *n* (%)
Region		
Tigray	217 (9.3)	530 (13.7)
Afar	56 (2.4)	74 (1.9)
Amhara	337 (14.5)	687 (17.8)
Oromia	294 (12.6)	458 (11.9)
Somalia	5 (0.2)	20 (0.5)
Benishangul-Gumuz	135 (5.8)	298 (7.7)
SSNP	239 (10.3)	541 (14.2)
Gambella	126 (5.4)	259 (6.7)
Harari	199 (8.5)	196 (5.1)
Addis Ababa	515 (22.1)	537 (13.9)
Dire Dawa	204 (8.8)	258 (6.7)
Age of respondent		
15–19	150 (6.4)	226 (6.9)
20–24	430 (18.5)	774 (20)
25–29	537 (23.1)	871 (22.6)
30–34	390 (16.8)	759 (19.7)
35–39	384 (16.5)	619 (16)
40–44	275 (11.8)	379 (9.8)
45–49	161 (6.9)	190 (4.9)
Residence		
Urban	1,247 (46.4)	1,496 (38.8)
Rural	1,080 (53.6)	2,362 (61.2)
Level of education		
No formal education	1,031 (44.3)	1,659 (43)
Primary school	542 (23.3)	1,297 (33.4)
Secondary	645 (27.7)	518 (13.4)
Diploma and higher	109 (4.7)	384 (9.9)
Marital status		
Married	1,787 (76.8)	3,078 (79.8)
Single/divorced/widowed	540 (23.2)	780 (20.2)
Wealth status		
Poor	273 (11.7)	976 (25.3)
Middle	276 (11.9)	588 (15.2)
Rich	1,778 (76.4)	2,294 (59.5)
Working status		
Yes	1,089 (46.8)	2,106 (54.6)
Not working	1,238 (53.2)	1,752 (45.4)
Religion		
Orthodox Christian	1,544 (66.3)	2,179 (56.5)
Muslim	466 (20)	876 (22.7)
Protestant	277 (11.9)	759 (19.7)
Other	40 (1.7)	44 (1.1)
Household head		
Male	1,739 (74.7)	2,947 (76.4)
Female	588 (25.3)	911 (23.6)
Number of children		
Less than 1	680 (29.2)	1,313 (34)
2–4	943 (40.5)	1,495 (38.8)
Above 4	704 (30.2)	1,050 (27.2)
Media exposure		
Yes	1,684 (72.4)	2,171 (56.3)
No	643 (27.6)	1,687 (43.7)

EDHS, Ethiopia Demography and Health Survey.

### 3.2. Maternal and reproductive characteristics

**Figure 1:**
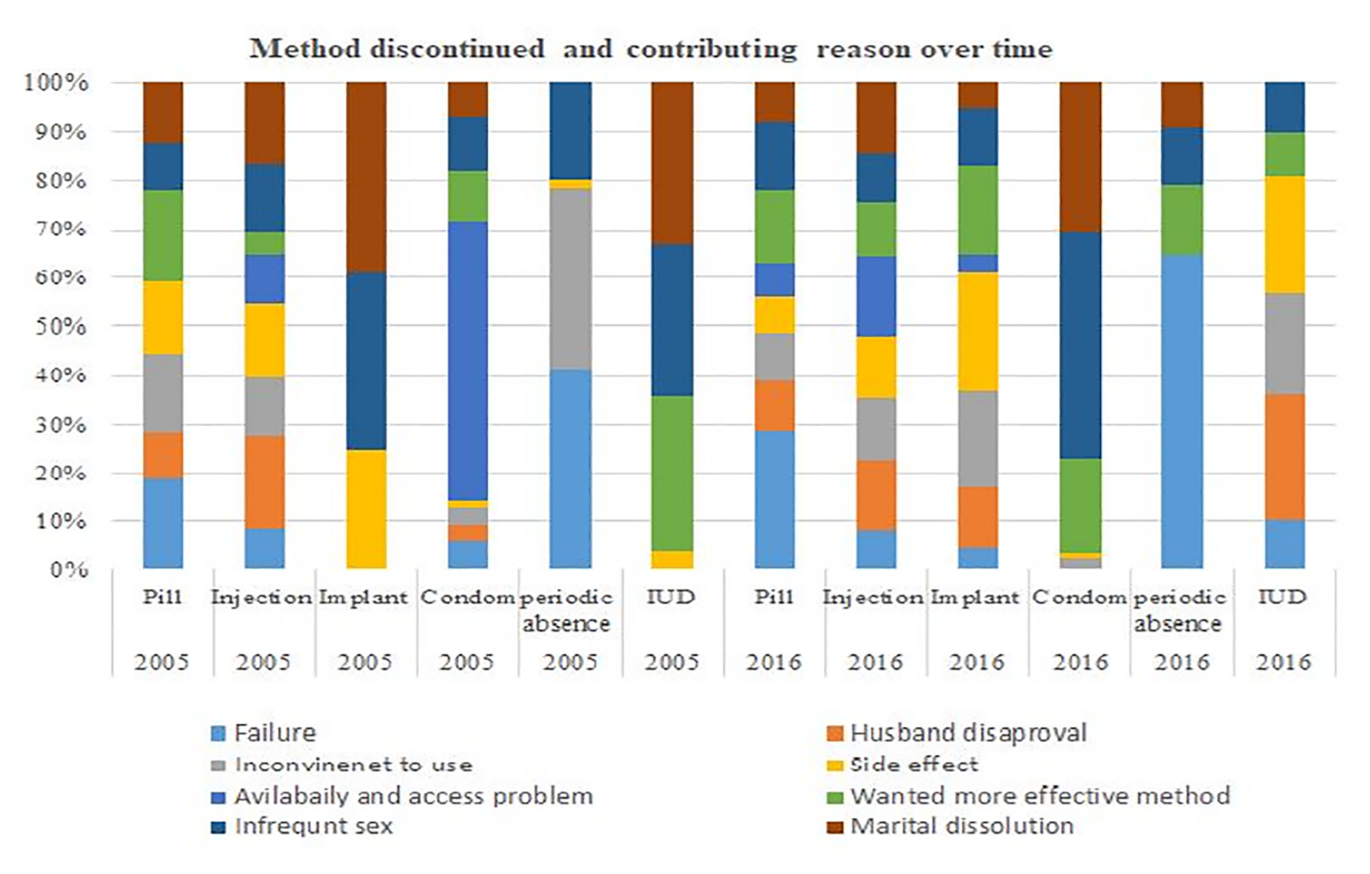
Graph showing the reasons for contraceptive discontinuation in Ethiopia in the years 2005 and 2016.

Approximately 11.1% of women in 2005 and 10.6% in 2016 had a history of termination of pregnancy. In addition, 57.6% in 2005 and 44% in 2016 were aware of family planning in the past month. With regard to unscheduled discontinuation methods, injectables and pills were the most common ones found in the two surveys. Side effects (40.8%), pregnancy (10%), and infrequent sex and separated husbands (7.3%) were found to be the most common reasons in the 2005 EDHS, whereas side effects (29.9%), infrequent sex and separated husbands (18.4%), and wanting a more effective method (5%) were found to be the most common reasons for discontinuation in the 2016 EDHS (Table 2) and (Figure 1).

**Table 2:** Maternal and reproductive characteristics of women in Ethiopia, 2005–2016.

Characteristics	EDHS 2005	EDHS 2016
History of termination of pregnancy		
Yes	258 (11.1)	411 (10.6)
No	2,069 (88.9)	3,447 (89.4)
Had ANC visit for the latest child		
Yes	747 (32.7)	1,749 (79.2)
No	1,580 (67.9)	459 (20.8)
Had postnatal follow-up		
Yes	43 (3.3)	298 (13.5)
No	1,294 (96.7)	1,910 (86.5)
Place of birth for the latest child		
Home	935 (69.1)	1,032 (46.7)
Health facility	402 (30.1)	1,176 (53.3)
Delivery by CS		
Yes	69 (5.2)	123 (5.6)
No	1,268 (94.8)	2,085 (94.4)
Was aware about family planning		
Yes	1,341 (57.6)	1,696 (44)
No	986 (42.4)	2,162 (56)
Reason for discontinuation	*n* = 910	*n* = 1,840
Became pregnant (failure)	91 (10)	120 (6.5)
Side effects	372 (40.8)	550 (29.9)
Husband disapproval	29 (3.2)	32 (1.7)
Access, availability	36 (3.9)	103 (5.6)
Wanted a more effective method to use	67 (7.3)	276 (15)
Incontinent to use	46 (5)	203 (11)
Infrequent sex and separation of husband	65 (7.1)	338 (18.4)
Marital dissolution	62 (6.8)	70 (3.8)
Other	132 (14.5)	148 (8)
Methods discontinued	*N* = 910	*N* = 1,840
Injectable	421 (46.3)	1,181 (64.2)
Pill	368 (40.4)	248 (13.5)
Condom	60 (6.6)	52 (2.8)
IUD	19 (2.1)	33 (1.8)
Implant	7 (0.7)	243 (13.2)
Periodic absence	24 (2.6)	24 (1.3)
Other	11 (1.2)	59 (3.2)

EDHS, Ethiopia Demography and Health Survey; ANC, antenatal care; CS, cesarean section; IUD, intrauterine device.

### 3.3. Spatial distribution of unscheduled contraceptive discontinuation

**Figure 2:**
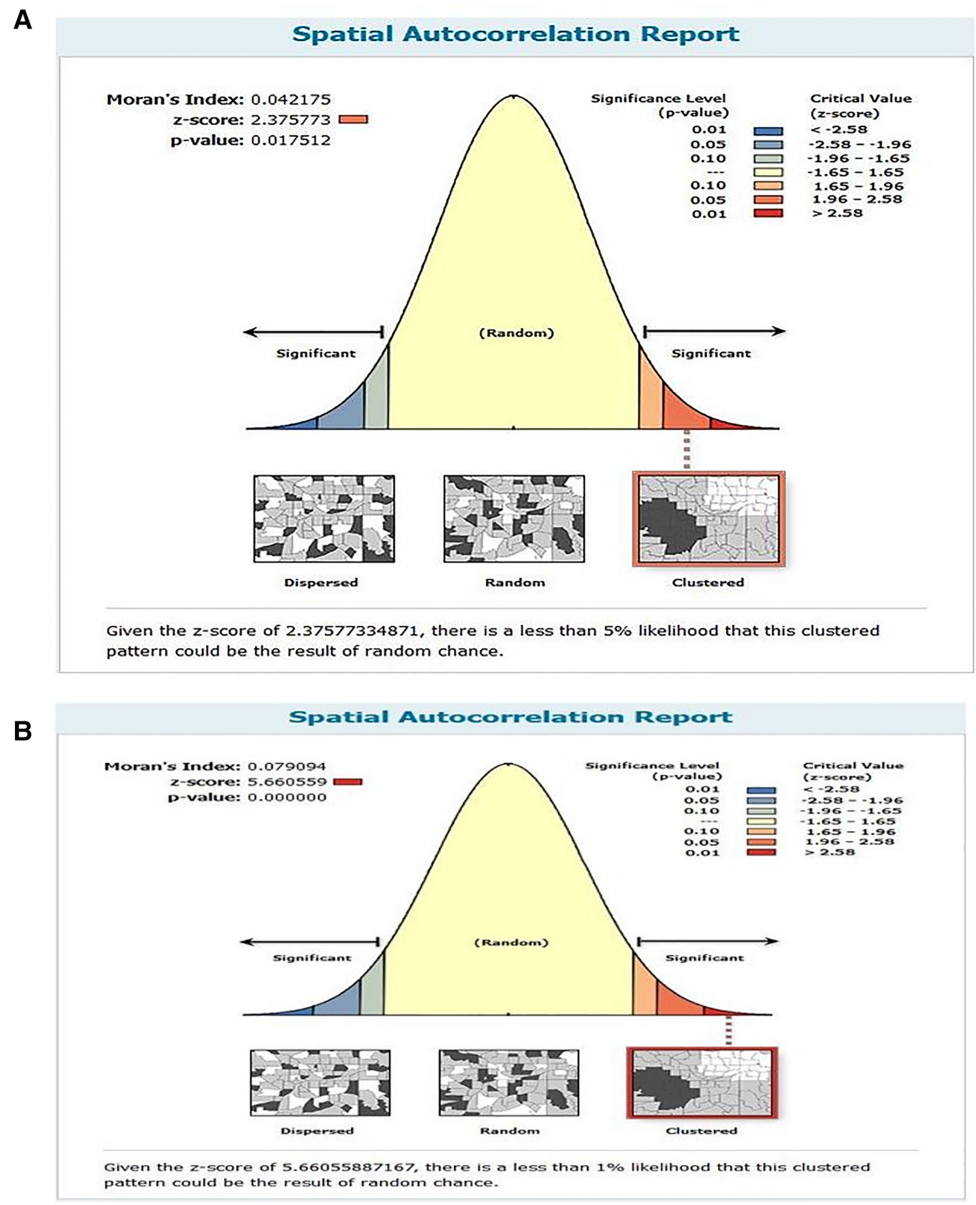
The global spatial autocorrelation of unscheduled contraceptive discontinuation in Ethiopia in (**A**) 2005 and (**B**) 2016.

The spatial distribution of unscheduled contraceptive discontinuation showed significant variations across the country over time (Figure 2). Global autocorrelation analysis showed the distribution of unscheduled contraceptive discontinuation. A high prevalence of clustering of unscheduled contraceptive discontinuation geographically aggregated in central Ethiopia, Addis Ababa, and eastern parts of the country like Dire Dawa, Central Amhara, and northern Tigray regions of Ethiopia in the year 2005.

**Figure 3:**
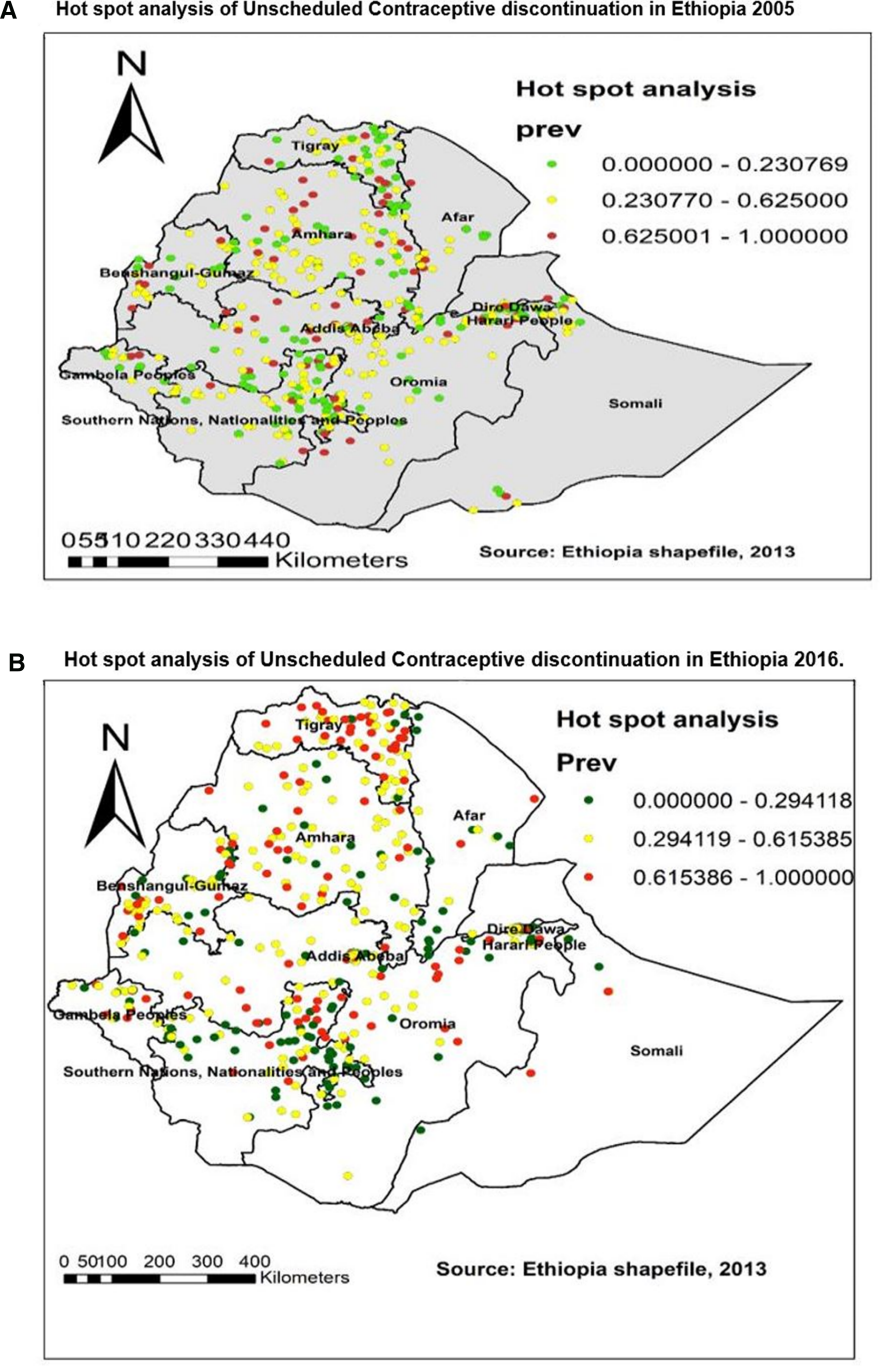
Hot spot analysis of unscheduled contraceptive discontinuation in Ethiopia in (**A**) 2005 and (**B**) 2016.

From the spatial analysis of 2005 EDHS data, a high prevalence of unscheduled contraceptive discontinuation was observed in Tigray, Southern Nations, Nationalities, and People’s Region (SNNPR), Benishangul-Gumuz, and Amhara region. Similarly, a high prevalence of unscheduled interruption of contraceptives was observed in Addis Ababa, Amhara, and Tigray regions and eastern Ethiopia, Harar, and Dire Dawa in the 2016 EDHS survey (Figure 3).

In 2005, a hot spot was detected in the eastern Amhara, Afar, and Oromia regions and some parts of the Southern regions of Ethiopia (Figure 3A). Similarly, in 2016, hot spots of unscheduled contraceptive discontinuation were detected in the northwestern parts of the Amhara, Addis Ababa, and Oromia regions (Figure 3B).

**Figure 4:**
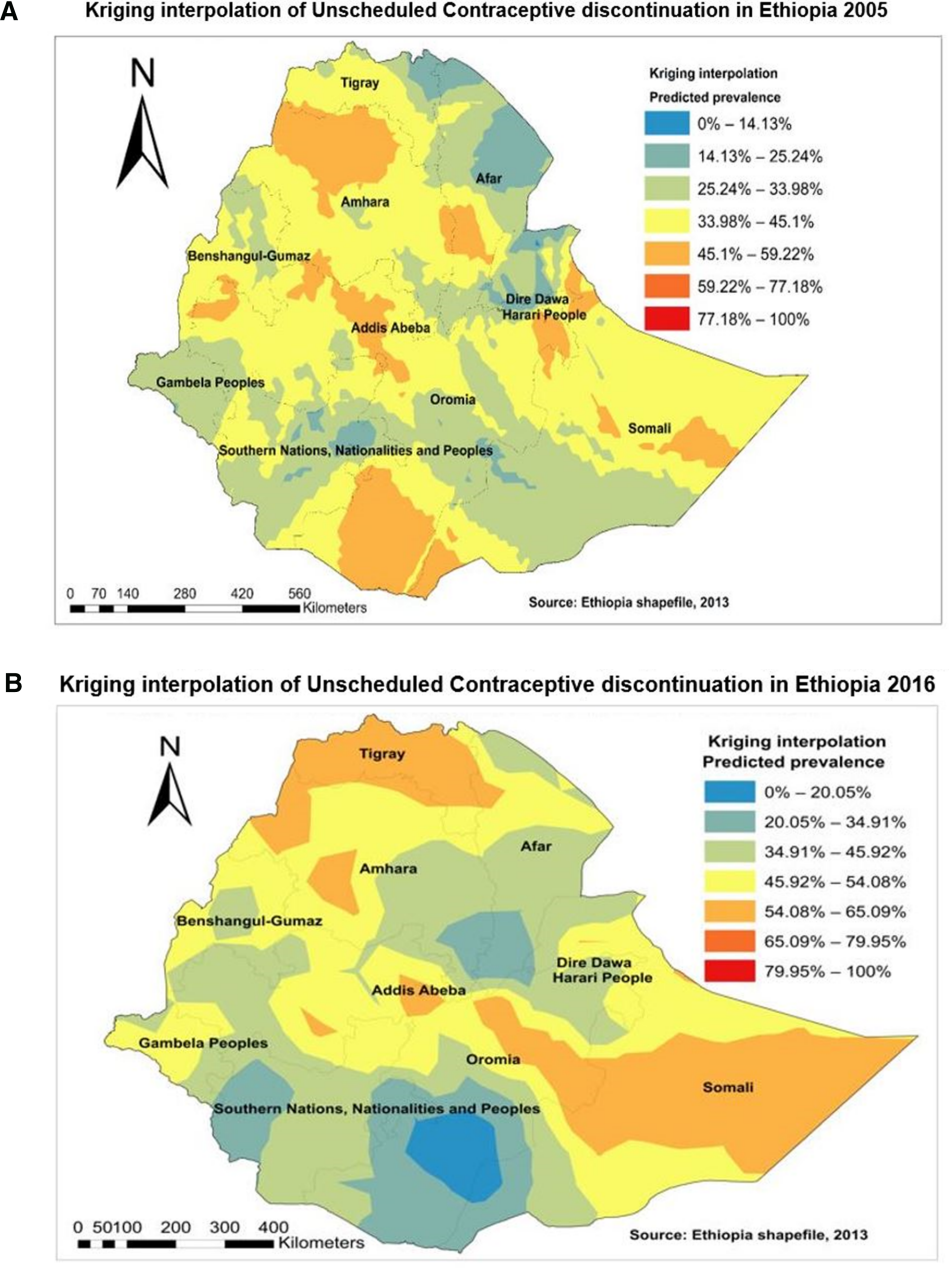
Kriging interpolation of unscheduled contraceptive discontinuation in Ethiopia in (**A**) 2005 and (**B**) 2016.

Based on 2005, Kriging interpolation predicted that the highest magnitude of unscheduled contraceptive discontinuation was found in Amhara, Afar, SNNPR, Oromia, Harari, and Dire Dawa. In contrast, a low prevalence of unscheduled discontinuation was found in the northeastern part of Afar, Dire Dawa, and SNNPR (Figure 4A). Likewise, according to the 2016 Kriging interpolation, the northern part of Tigray, Amhara, Oromia, and SNNPR regions and most of Somalia were predicted to have a high prevalence of unscheduled interruption of contraceptive use (Figure 4B).

### 3.4. Spatial SaTScan analysis of unscheduled contraceptive discontinuation—Bernoulli-based model

**Figure 5:**
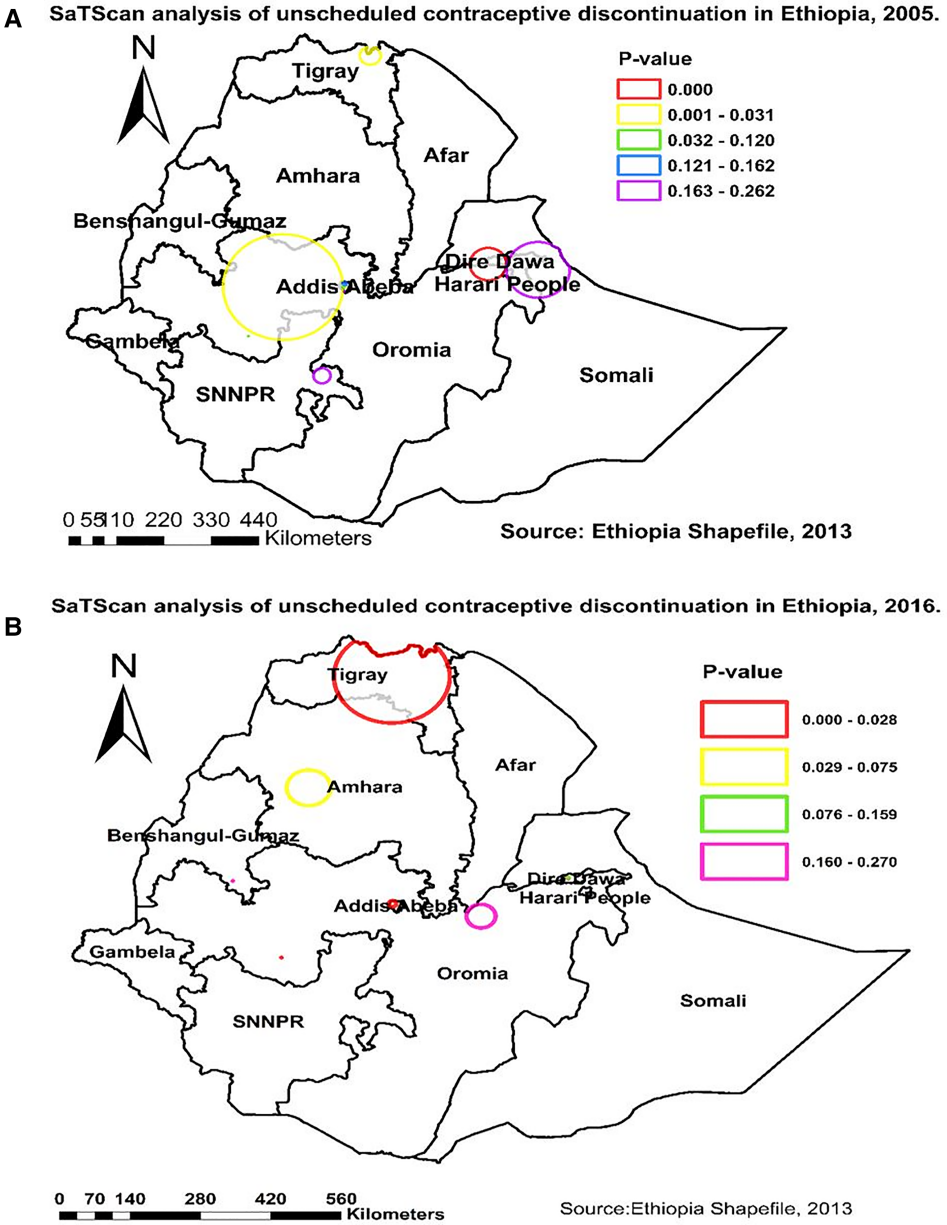
SaTScan analysis of unscheduled contraceptive discontinuation in Ethiopia in (**A**) 2005 and (**B**) 2016.

A spatial SaTScan analysis was done to identify significant clusters. Analysis results from the 2005 EDHS revealed that the primary clusters’ spatial windows were located in Northern Tigray, most parts of western Oromia, and some parts of the Southern Amhara and Dire Dawa regions. The window was centered at 9.501600N and 41.759800E within a radius of 41.86 km. Women who lived in this spatial window had 18.06 times higher risk of unscheduled contraceptive discontinuation compared with those who lived outside of this spatial window (Table 3, Figure 5A).

**Table 3A:** SatScan analysis of unscheduled contraceptive discontinuation in Ethiopia, 2005.

Cluster	Significant enumeration areas (clusters) detected	Coordinate/radius	Population	Case	RR	LLR	*P*-value
Primary	379, 425, 369, 371, 287, 196, 92, 166, 456, 38, 300, 121, 422, 91, 446, 79, 301, 173, 521, 473, 495, 85, 63, 321, 179, 233, 378, 314, 141, 169, 297, 386, 469, 294, 208, 306	9.501600N, 41.759800E, 41.86 km	237	48	18.06	50.016	0.001

LLR, log likelihood ratio; RR, relative risk.

**Table 3B:** SaTScan analysis of unscheduled contraceptive discontinuation in Ethiopia, 2016

Cluster	Significant Enumeration Areas (clusters) detected	Coordinate/radius	Population	Case	RR	LLR	P-value
Primary	636,156, 81, 590, 400, 597, 84, 551, 584, 579, 481, 355, 45, 181, 604, 461, 575, 340, 188, 98, 255, 479, 89, 528, 425, 430, 226, 129, 598, 538, 78, 404, 341, 583, 424, 94, 237, 550, 220, 623, 413, 605, 99, 196, 298, 268	13.954160N, 38.70663E, 111.62 km	583	180	1.39	22.15	<0.001
Secondary	100, 31, 107, 339, 626, 108, 11, 195, 314, 91, 635, 369, 59, 305, 487, 532, 645, 414, 159, 463, 274, 582, 608, 170, 144, 15, 110, 302, 153, 112, 293, 145, 225, 639, 247, 19, 464	9.025640N, 38.717480E 7.42 km	356	222	1.35	5.13	<0.001

LLR, log-likelihood ratio; RR, relative risk.

The same analysis was done using an EDHS 2016 dataset, and we found two spatial windows. The first window was a primary clusters’ spatial window that had 46 significant clusters and was located in most parts of Tigray and Southern Amhara regions. It was centered at 13.954160N and 38.70663E within a radius of 111.62 km. The other window was a secondary spatial cluster that was located in Addis Ababa within a radius of 7.42 km and had 37 significant clusters. Women who lived in the primary and secondary windows had 1.39 and 1.35 times higher risk of unscheduled contraceptive discontinuation, respectively, compared with those who lived outside of those spatial windows (Table 4, Figure 5B).

**Table 4:** Multilevel analysis of unscheduled contraceptive discontinuation in Ethiopia, 2016.

Characteristics	Mode I	Model II	Model III	Model IV
Age				
Less than 25		Ref		Ref
25–34 years		1.22 (0.98–1.53)		1.16 (0.92–1.46)
≥35 years		1.02 (0.76–1.37)		0.95 (0.71–1.28)
Sex of household				
Male				Ref
Female		0.86 (0.70–1.06)		0.82 (0.67–1.02)
Level of education				
No formal education		Ref		Ref
Primary		1.25 (1.02–1.53)		1.19 (0.97–1.46)
Secondary		1.37 (1.03–1.82)		1.25 (0.93–1.68)
Diploma and above		1.55 (1.13–2.14)		1.40 (1.01–1.95)*
The number of children				
≤1		Ref		Ref
2–4		1.44 (1.14–1.82)		1.46 (1.15–1.84)*
>4		1.25 (0.91–1.73)		1.34 (0.97–1.86)
Marital status				
Single		Ref		Ref
Married		10.32 (6.68–15.94)		10.79 (6.98–16.69)*
Separated/divorced		1.48 (1.03–2.13)		1.54 (1.07–2.30)*
Health access problem				
Yes		0.92 (0.77–1.08)		0.96 (0.81–1.14)
No		Ref		Ref
Heard about FP				
Yes		1.23 (1.02–1.48)		1.13 (0.93–1.38)
No		Ref		1
Ever-terminated pregnancy				
Yes		1.50 (1.16–1.95)		1.47 (1.14–1.91)*
No		Ref		
Involved in the decision-making of contraceptive use				
Yes		Ref		Ref
No		38.87 (28.56–52.90)		39.26 (28.84–53.45)*
Community-level variables				
Urban			1.34 (1.11–1.60)	1.37 (1.08–1.72)*
Rural			Ref	Ref
ICC	11.1	5.53	10.41	5.31
MOR	1.84 (1.78–1.89)	1.506 (1.38–1.66)	1.79 (1.62–1.90)	1.50 (1.45–1.53)
PCV		54.7%	7%	55.1%
Var	0.41 (0.36–0.45)	0.186 (0.11–0.28)	0.38 (0.25–0.46)	0.18 (0.15–0.20)
AIC				
Log-likelihood	−2,626.55	−2,043.72	−2,621.53	−2,040.06
Deviance	5,253.10	4,087.44	5,243.054	4,080.13

AIC, Akaike Information Criteria; FP, family planning; ICC, intraclass correlation; MOR, median odds ratio; PCV, proportion of change in variance.

* A statistically significant p-value of less than 0.05 in the regression analysis.

### 3.5. Determinants of unscheduled contraceptive discontinuation

From the multilevel analysis of the 2016 EDHS data, it was found that marital status, level of education, rural dwelling, involvement in the decision-making process, ever-terminated pregnancy, and the number of children were factors associated with unscheduled discontinuation of contraceptives. For those women who were married and separated or divorced, the odds of unscheduled discontinuation of contraceptives were 10.79 (AOR = 10.79, 95% CI: 6.98–16.69) and 1.54 (AOR = 1.54, 95% CI: 1.07–2.30) times higher than those of single women. Women who had attended secondary and above levels of education were associated with 1.40 (AOR = 1.40, 95% CI: 1.01–1.95) times higher than those who had no formal education. Likewise, women who had a history of termination of pregnancy were associated with unscheduled discontinuation of contraceptives compared with those with no formal education. Similarly, for women who had 2–4 children, the odds of unscheduled discontinuation were 1.46 (AOR = 1.46, 95% CI: 1.15–1.84) times higher than those who had less than one child. Women who were not involved in the decision-making process of contraceptive use had 39.26 (AOR =  39.26, 95% CI: 28.84–53.45) times higher odds of unscheduled discontinuation compared with those involved in the decision-making process. Urban-dwelling women have been associated with increased odds of unscheduled contraceptive discontinuation than women from rural areas (AOR = 1.37, 95% CI: 1.08–1.72). Furthermore, the MOR indicates that the unscheduled contraceptive discontinuation in the null model was 1.84, which shows there was a significant variation between enumeration areas (clustering). In the full model, after accounting for all factors, the MOR decreased to 1.50, which explains the presence of the clustering effect in the full model (Table 3).

## 4. Discussion

This study showed that the pattern of unscheduled discontinuation of contraceptives increased from 39.1% in 2005 to 47.7% in 2016. Injectables, pills, and implants were the most common methods of contraceptives used, as found in the 2005 and 2016 EDHSs. In 2005, side effects, contraceptive failure, infrequent sex, and marital dissolution were found to be the most common reasons for the unscheduled discontinuation of contraceptives. Similarly, in 2016, EDHS, side effects, infrequent sex, and wanting more effective methods were found to be the most common reason for unscheduled contraceptive discontinuation in Ethiopia. In general, the magnitude of unscheduled discontinuation increased over time, which could be attributed to side effects, increased fertility intentions, and misconceptions about contraceptives in the community (12).

This study also revealed that there was a clustering or non-random distribution of unscheduled contraceptive distribution or significant spatial variations (clustering) at regional levels of Ethiopia, as found in the two surveys (2005 and 2016). On the other hand, the hot spots of the problem (unscheduled contraceptive discontinuation) were located in the eastern Amhara, Afar, and Oromia regions and some parts of the Southern regions of Ethiopia. This finding was supported by previous study findings in Ethiopia and Kenya, where discontinuation was higher in the urban areas of the country (4). In the urban areas, most reproductive-age women used short-acting contraceptives. An interpolation of this study indicated that parts of Afar, Amhara, Oromia, Somalia, Harari, and Dire Dawa had a high prevalence of unscheduled discontinuation of contraceptives. The predicted high prevalence of unscheduled discontinuation of contraceptives was also observed in the majority of the Somali and Tigray regions and some parts of the Oromia and Amhara regions. This finding was consistent with the previous study findings in Ethiopia (3, 15, 16). In addition, these geographical variations could be attributed to sociodemographic, cultural taboos, and healthcare accessibility problems. In addition, those interpolated areas are emerging regions with weak and unorganized health systems to meet the needs of the community. Moreover, these areas are recognized by the Ministry of Health as the most disadvantaged areas and have high maternal mortality from preventable causes (17, 18).

In this study, individual levels of education, the number of children, marital status, history of termination of pregnancy, and involvement in the decision-making process of contraceptive use were identified as significant determinants of unscheduled discontinuation of contraceptives. Thus, separated/divorced women were associated with increased odds of unscheduled contractive discontinuation than singles. This could be attributed to infrequent sexual activity after marital dissolution. On the other hand, women who were married were associated with increased odds of contraceptive discontinuation compared with singles. This finding was consistent with previous studies (12). This could be attributed to increased fertility intentions and having the desired number of children. Thus, the problem of unscheduled discontinuation of contraceptives among married women might be overcome by male engagement and enhancement of couple communication (19). Similarly, women who had two to four children were associated with increased odds of unplanned discontinuation compared with those who had only one or no child. This could be attributed to women desiring to have more children compared with those desiring to have only one child. This finding was consistent with that of previous studies (4, 8, 20).

In addition, those who attended secondary education were more likely to experience unscheduled contraceptive discontinuation compared with those who had no formal education. The reason for this could be that women who had some level of education may have a better understanding of the undesirable health effects of contraceptives. Short-acting contraceptives are hormones that have systemic effects such as weight gain and increased risk of diabetes and heart disease. Likewise, women who had a history of termination of pregnancy were associated with unscheduled discontinuation of contraceptives compared with those with no formal education. The reason for this could be that women who discontinued contraceptives may have had unwanted and unplanned pregnancies that forced them to terminate their pregnancies (5). This study finding is supported by that of other studies in Kenya (4). Similarly, women who had —two to four children had higher odds of unscheduled discontinuation than those with one child. This finding was consistent with that of previous studies (12, 15). Involvement in the contraceptive decision-making process has been strongly associated with unscheduled discontinuation of contraceptives. This finding was consistent with that of previous findings (12, 17, 20). This involvement in the decision-making process is an important issue that needs to be addressed by multisectoral interventions so that the decision-making ability of women could be improved, because male hegemony is something that is traditionally and culturally accepted. This hegemony can be broken by improving women’s socioeconomic status and increasing community awareness. Urban-dwelling women have been associated with increased odds of unscheduled contraceptive discontinuation than those from rural areas. This finding was consistent with that of previous studies in Ethiopia and Kenya (4, 6, 12, 15, 21). Women who reside in urban areas tend to use short-term methods such as emergency contraceptives (22). However, the advantage in urban areas is the availability of health services that help in switching and shifting of methods more frequently than in rural areas (3).

This study has implications for reproductive-age women, policymakers, and healthcare administrators to unfold the reasons for unscheduled discontinuation of contraceptive methods. In addition, this study is expected to play a pivotal role in the easy identification of intervention areas and efficient utilization of resources.

This study was based on nationally representative data that are expected to improve the generalizability of its findings. In addition, spatial distribution has great importance for the efficient allocation of scarce resources. However, this study shares the limitations of a cross-sectional study, as exemplified by the fact that health system factors were not available and not accounted for in the multilevel analysis.

## 5. Conclusion

This study revealed that unscheduled discontinuation of contraceptive distribution was significantly clustered in central, north, and eastern parts of Ethiopia, as found in two surveys. The magnitude of unscheduled contraceptive discontinuation increased from the year 2005 to 2016. The finding underscores the need for further interventions such as the use of multiple mixed methods and improvement in women's decision-making ability in the choice and use of contraceptive methods in hotspot areas of Ethiopia.

## Data Availability

Publicly available datasets were analyzed in this study. These can be found here: www.dhsprogram.com.
